# The case of CAUSE: neurobiological mechanisms for grounding an abstract concept

**DOI:** 10.1098/rstb.2017.0129

**Published:** 2018-06-18

**Authors:** Friedemann Pulvermüller

**Affiliations:** 1 Brain Language Laboratory, Department of Philosophy and Humanities, WE4, Freie Universität Berlin, 14195 Berlin, Germany; 2 Berlin School of Mind and Brain, Humboldt-Universität zu Berlin, 10099 Berlin, Germany; 3 Einstein Center for Neurosciences Berlin, Charitéplatz 1, 10117 Berlin, Germany

**Keywords:** causation, distributed neuronal circuit, embodiment, grounded cognition, Hebbian learning, semantics

## Abstract

How can we understand causal relationships and how can we understand words such as ‘cause’? Some theorists assume that the underlying abstract concept is given to us, and that perceptual correlation provides the relevant hints towards inferring causation from perceived real-life events. A different approach emphasizes the role of actions and their typical consequences for the emergence of the concept of causation and the application of the related term. A model of causation is proposed that highlights the *family resemblance* between causal actions and postulates that symbols are necessary for *binding together the different partially shared semantic features of subsets* of causal actions and their goals. Linguistic symbols are proposed to play a key role in binding the different subsets of semantic features of the abstract concept. The model is spelt out at the neuromechanistic level of distributed cortical circuits and the cognitive functions they carry. The model is discussed in light of behavioural and neuroscience evidence, and questions for future research are highlighted. In sum, taking causation as a concrete example, I argue that abstract concepts and words can be learnt and grounded in real-life interaction, and that the neurobiological mechanisms realizing such abstract semantic grounding are within our grasp.

This article is part of the theme issue ‘Varieties of abstract concepts: development, use and representation in the brain'.

## 1. Introduction: the aboutness of symbols and semantic grounding

**Figure 1.**
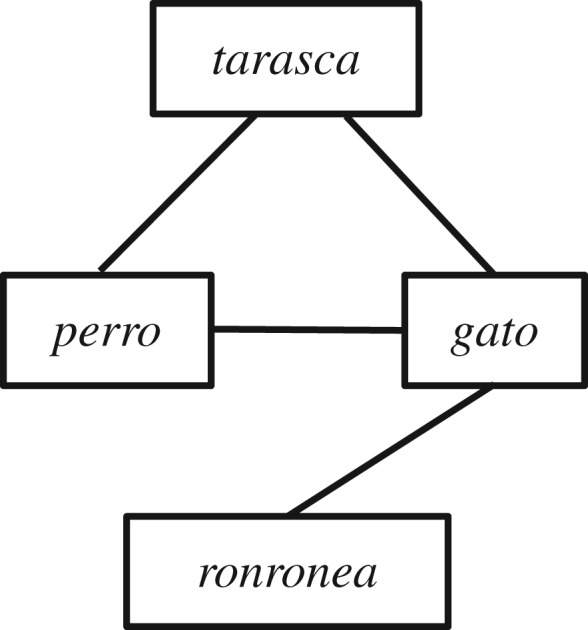
Semantic network for four word forms. Although the lines indicate semantic relationships between words, the diagram does not provide sufficient information for word understanding. For discussion, see text.

Assume you want to learn a new language and a nice fellow tells you that ‘perro', ‘gato', ‘tarasca' and ‘ronronea' are all words of that language. Suppose that, furthermore, you are told that the first three words are closely related to each other with regard to their meaning, whereas the last word only relates to the second one (figure 1). Would this information allow you to understand these words? Probably not—although note that you have been provided with semantic, that is, meaning-related, information. As this information seems insufficient for understanding, the nice chap would give you more detailed (distributional semantic) information by saying that a ‘gato' would occasionally ‘ronronea', whereas a ‘perro' might possibly ‘tarasca' the ‘gato'. He could even tell you explicitly the lexical category of these words (e.g. noun, verb). Now, given these hints, would you be able to understand? It is clear that still much more is needed for understanding the meaning of new symbols or to make these symbols (fully) interpretable.

This simple example allows one to rule out a whole class of cognitive models of semantics as insufficient for explaining meaning. These are models defining meaning exclusively in terms of relationships between symbols. It does not matter so much in this context whether between-symbol links are set up by verbal explanation (in the same language), by lines between symbols in connected graphs (as in figure 1, see, e.g. [1]) or by semantic vectors coding the co-occurrence of symbols in large collections of texts [2]. These approaches lack an important semantic component, which connects the symbols to the entities they are used to speak *about*. ‘Symbols' lacking this *aboutness* information are not interpretable; therefore, they are not symbols at all. To explain how symbols relate to what they are about is sometimes seen as a problem, called the *symbol grounding problem* [3]. Some still doubt that there is anything to be explained here (e.g. [4,5]); therefore, this introduction is given.

However, in many cases, help in grounding symbols is easy to provide. By pointing to objects, the majority of the words in most languages can be explained to a degree. ‘Perro' can be explained by showing pictures of a bulldog, a German shepherd and a boxer, and ‘gato' by pointing to a British shorthair, a Maine coon and Siamese kitten. Pointing to ‘things' is not always an option because not all words relate to real-world objects [6]. For example, a significant number are used to speak about actions. For these, the semantic explanation may embed bodily actions. One could make a certain sound and tell the language learner ‘that's ronronea(r)', or wait until the learner would bite into an apple to ‘semantically ground' ‘tarasca(r)'. Most people will understand now, at least approximately, although these explanations lack precision and are prone to errors, for example to mistaking a fox for a ‘perro', a tiger cub for a ‘gato' or high table dining for ‘tarascar'.

Note that a single grounding event (relating a word to (the picture of) a Siamese cat) is normally not sufficient, because too many degrees of freedom are still available (is it the fur, tail, eyes, ears or entire gestalt the word relates to?—see [7]). Repeated word use in the presence of different referent objects allows the learner to extract the relevant shared semantic features from the percepts and to build abstract conceptual representations ignoring some of the peculiarities of the individual instances. The grounding framework suggests that at least some concepts (e.g. GREEN) can be explained by *perceptual and action-related feature extraction* from concrete perceptions and actions (the greenness of the cucumber, avocado and crocodile). The brain seems well prepared for such feature extraction, because it is equipped with powerful mechanisms for extracting and storing correlations in neural activity (for review, see [8,9]). Note, furthermore, that not each and every word needs to be semantically grounded in experiences. Given a number of words are grounded directly in objects, actions and real-life events, it is possible to transfer this knowledge to new symbols—by explaining, using grounded symbols, what the new ones are about, or by semantic feature extraction from distributional information across contexts. Given a base vocabulary of semantically grounded symbols is available, other similar items can be learned verbally, so that graphs and vectors indicating degrees of semantic similarity become useful [3,10]. However, such grounding transfer or *indirect grounding* only works if there is a close semantic relationship between already known and new items. By using visually grounded words such as ‘horse' and ‘stripes', the related item ‘zebra' can be verbally explained [3], but not ‘guilt', ‘love' or ‘therefore'. It appears that, for each semantic domain, at least a few vocabulary items need their own direct grounding in order to allow similar items to be verbally explained and taught.

## 2. Aboutness and grounding of abstract words

While pointing to referent objects and actions provides reasonable clues for learning what symbols are used to speak about, it is questionable whether this strategy is applicable to other semantic symbol types, especially those normal speakers tend to classify as abstract in meaning [11,12]. First, consider abstract words related to feelings, emotions and other ‘internal states' of the body, such as ‘love' and ‘desire'. Some researchers suggest that ‘grounding in emotion and internal states’ is as straightforward as relating words to visible referent objects (see, for example, [13]). However, there is an important difference: The internal state cannot be perceived by others, as it is an ‘inner' feature of only one person. Therefore, the typical basis of grounding, the common reference object shared between learner and teacher, is missing. Suppose our learner had to find out whether ‘enojoso' means a positive, enjoyable and calm inner state or rather one of discontent, grumpiness, even angriness. How would the teacher go about making sure the learner does not ‘internally label' the wrong feeling? Semantic grounding can only succeed in social interaction if *shared criteria* are available for correct symbol application, and the main criterion for correct application of a word like ‘gato'—that is, its relationship to appropriate reference objects—is not applicable to inner states if they are not manifest in the ‘outer' social world shared between individuals. The solution comes from the bodily ‘symptoms' indicating specific feelings, the body actions that express these ‘inner' states [6,14,15]. Therefore, a straightforward avenue towards teaching the meaning of ‘enojoso' is to apply the word when the learner performs actions that satisfy the criteria for enojoso-ness—be they a happy or angry face, a relaxed or tight posture, the careful stroking of an object or an aggressive gesture towards it. To learn what emotion words (and other symbols related to psychological states) are about, inner state expression in action is required. Therefore, ‘grounding in emotion' is grounding in action.

In line with this view, motor systems of the human cortex activate in emotion language processing [16,17]. Important clues for the relevance of motor mechanisms for emotion semantics come from autistic persons with motor dysfunction and deficits in expressing their emotions. These subjects show concordant processing abnormalities for action- and emotion-related words; when processing abstract emotion words such as ‘love’ and ‘disgust’, they do not activate their motor system and their degree of underactivity in the motor system indicates their level of autistic traits [18]. The relationship between motor underactivity and conceptual-linguistic abnormalities is consistent with the proposal that the cortical motor system and the action-related information it processes are essential for semantic grounding of abstract emotion-related symbols. For other abstract words, which are used to speak about non-emotional inner states (for example, ‘mental' words like ‘thought' or ‘idea’), a similar grounding pathway may be possible, although current data addressing this issue are open to several different interpretations (see [19,20]).

A second class of abstract symbols, for which questions may arise with regard to their semantic grounding, includes items such as ‘beauty' or ‘democracy'. Given these terms are applied consistently, they are, used to speak about concrete sets of entities—of objects or systems of government, respectively—although their grounding heavily draws on abstraction abilities. Whereas concrete words are used to speak about a narrowly defined range of objects, actions, scenes or modes of interaction, abstract symbols can be used to speak about rather different such entities [21–23]. In this sense, the concrete expression ‘Siamese cat' is narrower in its use than the general and thus more abstract terms ‘cat’ and ‘animal', and for the highly abstract symbols ‘beauty' and ‘democracy' the range of possible instances may even be broader. However, this variability creates a problem for explaining the semantic links of abstract terms. There is little hope to explain the semantic links of highly abstract terms by associations between symbols and broad selections of real-life entities. One reason for this comes from neurobiologically founded correlation learning mechanisms, which imply association of co-occurring entities but likewise dissociation and weakening of connections between representations that activate independently from each other [8]. This makes it difficult to ‘associate' a wide range of diverse sensorimotor exemplars with the same word, as the word would rarely associate with, but frequently dissociate from each of its instances, thus yielding a net effect of leaving the abstract word's form circuit semantically unconnected. It has been suggested that the learning problem can be circumvented by indirect grounding—through association of abstract words with concrete words, which have previously been grounded directly. Such indirect grounding can indeed work if the abstract items are primarily *associated with a small set* of other words (for a model exploiting this idea, see [23]), And indeed, some data indicate that the learning of abstract words relies less on direct grounding in experiences than on verbal contexts and indirect grounding (see, for example, [24]). However, if abstract words appear in more variable non-linguistic contexts, it seems plausible that their linguistic placement in text contexts is also more variable, a hypothesis in part supported by studies assessing the variability of the first semantically related words that come to mind when encountering concrete and abstract items [25,26]. Greater variability of contextual embedding and real-word grounding comes with the dissociation problem, that neurons out-of-sync delink, so that the neural representations of very variable instances and contexts of an abstract word may become difficult to bind to those of the symbol's form.

A solution for this abstract binding problem can be offered if the role of semantic feature structure is taken into account. For concrete terms, relevant semantic features are shared between most or all instances they are typically used to speak about. By contrast, the meaning of an abstract term is not sufficiently explained by a set of semantic features shared by all of its instances, because such a shared set is typically missing. Relevant features may not apply to all cases to which the term is normally applied, but only to some of them. Still, *family resemblance*—sharing of *some* features typical of *subsets* of the category [6,27]—applies to all instances, so that the partially shared features can be used to build the highly abstract concept. The partially shared features would frequently activate in conjunction with the word form, so that associative correlation-based learning can apply at the level of their neuronal representations. This leads to an extension of the grounding concept by semantic feature extraction, conjunction and disjunction mechanisms (see [28]). Figure 2 contrasts the semantic overlap pattern that may account for typical concrete words—including an intersection of semantic feature neurons—with a family resemblance pattern postulated for abstract items—which includes partially overlapping neuronal sets. For both concrete and highly abstract terms, ‘semantic anchor neurons' connect word form and instances the word is used to speak about. The semantic connections are stronger for concrete than for abstract words, explaining the greater difficulty to recall concrete word-related events for the latter [29]. The greater variability of instances of abstract words is captured by the family resemblance pattern of partial overlap. The feature-based analysis is enforced by neurobiological mechanisms.

**Figure 2.**
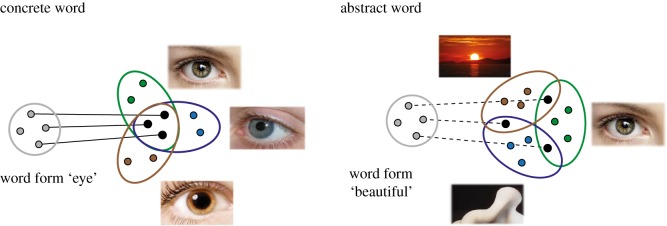
Different semantic structures and neurobiological mechanisms for concrete and abstract words. (Left panel) The concrete word ‘eye' is used to speak about objects with similar shapes and a range of colours. At the neurocognitive level, this leads to exemplar representations that strongly overlap in their sensorimotor semantic feature neurons (overlapping sets). The panel shows ‘semantic anchor neurons' in the intersection of sets and feature neurons more specific to individual exemplars. In concrete semantic learning, neurons of the circuit that overlap strongly interlink with the word form circuit owing to high correlation of their activations. (Right panel) The instantiations of abstract words such as ‘beauty' are quite variable, exhibiting a family resemblance pattern of partial semantic similarity. The panel shows the putative neural correlate of such family resemblance as partially overlapping neuronal sets, where sensorimotor semantic anchor neurons are only shared between subsets of exemplar representations of variable instantiations of the concept. The low correlation of activations of neuronal circuits for word forms and for each exemplar representation results in weak links between neural representations of sensorimotor knowledge and those of verbal symbols. (Reprinted with permission from [28].)

A third group of abstract concepts might even lack any partial feature-related relationship to concrete entities in the world. Logical expressions such as ‘and', ‘or' or ‘not' are of this sort, and likewise the concepts of necessity, functional connectivity and causation, which are fundamental to our thinking and mental life. For these ‘distant' abstract items, it seems implausible to many that the knowledge about their meaning can be derived from experiences.

## 3. Causation as an inborn concept

Cognitive scientists typically believe that ‘distant' abstract concepts are *a priori* present in our minds. Neuroscientists have even argued that the meaning of some logical concepts is given to us by way of functional properties of the nervous system, by a logical calculus of ideas immanent in nervous activity [30]. In this spirit, a major review of causal thinking suggests that ‘animals, including human beings, may have some hard-wired representations that automatically specify that particular types of events lead to other events' [31], and semanticists postulate a universal concept, called CAUSE, which supposedly captures various different kinds of relationships between antecedents and their effects [32]. This perspective tries to unify a broad range of cases of causation and superficially-similar relationships, some of which, however, differ fundamentally from each other for example true causal effects as determined by physical laws and the (non-causal) interplay between human actions guided by intentions, assumptions and social norms [33]. Focusing on interactions between objects, Talmy distinguished variants of causation based on an analysis of finer primitives (force dynamic patterns), placing ‘cause' and ‘make' in a structured semantic neighbourhood with ‘helping', ‘letting', ‘hindering', ‘preventing' and so on [34].

Note that even if the concepts AND, OR and (variants of) CAUSE were inborn, there would still be a significant grounding problem. It would need to be shown how the brain-immanent neurocognitive circuits mechanistically organizing the concept might link up with real-life examples (for related suggestions, see [21,35]). This is not a trivial task, but one for which the mechanistic details would need to be worked out.

I will take a different avenue here. By taking a close look at the concept of causation, I will argue that this concept may in fact result from experiences, given that neurobiological mechanisms with sophisticated information processing capabilities are available.

## 4. Learning causal links by perceptual correlation

It seems obvious that causation is closely related to experiences. The billiard ball hitting another one, which then moves in a predictable direction, is a straightforward example [36]. Children may observe this pattern repeatedly and draw conclusions on causality. However, it is clear that correlation does not always imply causality—bird migration does not cause trees to lose their leaves, although the two occur in succession in many places. Still, Hume claims that ‘our idea … of the necessity and causality arises entirely from the uniformity, observable in nature; where similar objects are constantly conjoined together, and *the mind is determined by custom to infer the one from the appearance of the other*. … Beyond the constant conjunction of similar objects, and the consequent inference from one to the other, we have no notion of any necessity, or connexion' (§8.5 in [36]). Why should this be? According to Hume, causal links exist not in reality but only in our thoughts (or cognitive system) and perceptual correlation is sufficient to motivate the conclusion—although the conclusion may be false (as in the bird migration case). This position is consistent with an *a priori* predisposition to infer causality from perceptual correlation.

**Figure 3.**
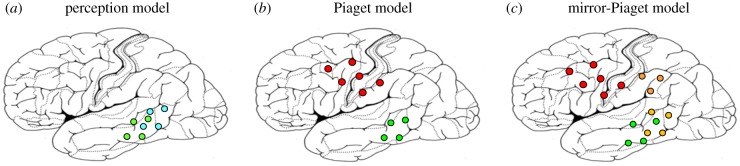
Different theories of causal learning and representation put emphasis on correlations between perceptions (perception model), or on the learning of motor acts and their typical perceptual consequences, which can become the goals of the acts (Piaget model). Further development of the Piaget model incorporates perception mechanisms of the motor act itself, thus interlinking it with the motor act representation (mirror-Piaget model). At the neurobiological level, causal links would thus be present (*a*) between perceptual representations in the temporal cortex (green and blue neural elements in the perception model), (*b*) between frontal motor act-related and temporal perceptual goal-related representations (connections between the frontal and posterior cortical regions, Piaget model), or (*c*) between motor and perceptual action representations in the frontal and posterior cortex (red and orange neural elements) and posterior perceptual goal representations (in green, mirror-Piaget model). Only the mirror-Piaget model explains causal learning by observation along with the ‘action advantage' in causal learning (see text for discussion).

Experimental research in infants and adults indicates that the correlation structure of events—that is, the pattern of co-occurrences between elementary actions—provides significant clues for grouping them together into larger units [37–39] and for interpreting them as causally linked [40]. This research is reminiscent of perceptual learning in the domain of language [41] and emphasizes the role of passive mapping in early learning. The results suggest that passive perceptual processing of sequences of perceptions links together the correlated perceptual representations. If elementary motion events are linked with each other, underpinning neurophysiological processes are probably situated in the posterior temporal cortex, in the human analogue of movement processing area MT. A neurobiological model of the causal link between two visual–perceptual movement representations is shown in figure 3*a*. In this model, a perceptual representation (e.g. birds migrating) links up with a second one (leaves falling), which the former predicts. The correlated represented entities would be seen as causally linked (in this case falsely).

## 5. A role of human action in learning causality?

In an entirely different tradition, Piaget proposed that learning of causality arises from an infant's own body actions [42,43]. Supposedly, the child starts to see a causal link between an intentional action and its perceived immediate effect in the world, for example the motor movement towards a toy and the consequent movement of the object, typically a ‘baby mobile' (for related data, see, for example, [44]). Recent studies in infants using ‘sticky mittens', by which babies still unable to grasp can manipulate objects, indeed indicate an influence of own body actions on learning the causal link between such actions and their perceived predictable consequences [45–47].

The neurobiological implementation of Piaget's model is very different from the perceptual learning model. Instead of associations between perceptual representations in the temporal cortex, the crucial causal link is organized as a fronto-temporal connection between neuronal motor programme and predictable perceptual representation (figure 3*b*). The action representation (hand/arm away movement) is interlinked with a perceptual schema (baby mobile moving), the typical action *goal*. Activation of the representation of the motor act leads to the pre-activation of the perceptual goal schema, which provides a mechanism for *forward prediction* of action-related perceptions (cf. feed-forward models of motor planning, e.g. [48]). Causality connects act and goal, and the fronto-temporal neural link maps and cognitively represents this causal link.

Piaget's approach has been criticized for several reasons: It only applies to immediate consequences between motion and adjacent objects the child is directly acting upon. Causal relationships frequently concern actions and their effects on distant objects far from the body (operating the switch and seeing the bulb light up). Crucially, learning of causal links can not only arise from own body actions but likewise from observing others perform an action and consequent changes in the environment. The neurobiological model following Piaget does not distinguish between near and distant target objects and thus has no difficulty capturing the distant object problem. Bridging a temporal gap between a motor act and effect can be implemented if the connected representations are allowed to maintain their activity for a while, which is very plausible if these are conceptualized as cortical neuron circuits [49,50]. However, an extension is necessary to account for causal learning by action observation (see, for example, [51]). It needs to be seen that, over and above knowledge about motor movements, knowledge about a motor act includes perceptual knowledge too, thus linking, for example, the motor programme for the arm and hand movement with the (visual and somatosensory) perception of the trajectory of this movement. So the perception of the motor act itself, the *basic action* of moving a part of the body [52], needs to be distinguished from the perception of the predictable action consequences, the *action goals*. Much recent research has shown that cortical mechanisms tightly interconnect motor and perceptual representations—both at the level of basic actions and at that of goal-directed ones, and that neurons in specific premotor and parietal areas can similarly activate in action performance and perception [53,54].

Replacing the motor representation of the motor act in the Piagetian model of figure 3*b* by a multimodal action–perception circuit, comprising neurons in motor, premotor and adjacent prefrontal cortex along with parietal and middle temporal cortex, leads to the ‘mirror-Piaget model' (figure 3*c*). Here, the basic action (hand/arm away movement) links up with two types of perceptual representations, for the motor act itself (hand moving) and for the predicted consequence (baby mobile moving).

As this model now captures the learner's knowledge about both own action (motor) performance and about perceptual aspects of motor acts, it can be employed for explaining the activation of causal action schemas not only by one's own actions but by the perception or recognition of other persons' actions too. The distributed action representation is activated—either by motor activity or by perceptual input during action observation—and, in both cases, is followed by that of the perceptual posterior temporal circuit processing the action's goal—with a causal neuronal link in the brain mapping the causal situation in the real world. Owing to neurobiological principles, the neuronal connection joining together knowledge about the action and its consequences will only be strengthened if there is a good correlation between them [55,56]. Incidental consequences or accidental co-occurrence of a percept would, therefore, be ineffective; only the typical consequences of the action, the action goals, come into play and are permanently stored by the emerging circuit. Note, furthermore, that the fronto-posterior connections implementing the causal link will be strengthened when the action precedes its consequence in time, but strengthen too if they occur simultaneously (as in the case of the light switch—for discussion, see [33]), because of the delay between motor and consequent perceptual activation.

The mirror-Piaget model captures both the learning by own action performance, plus observing its (local or distant) consequences, and the learning by observation of other people's actions, too. It also can be used to address non-standard causal action types, for example preventing an apple from falling by holding it tight (where no change in state is induced; see [34]). This model may be seen as a neurobiological underpinning of aspects of interventionist accounts of causality, which emphasize the fact that causes are potential means for manipulating their effects [57–59].

One may question the applicability of this model to Hume's billiard balls, as in this case, the antecedent is not a human action but just a (perceived) rolling ball. However, following the interactionist mirror-Piaget model, the human action, which is the typical antecedent of the balls rolling, would come into play by way of a ‘mirror' mechanism, thus activating an antecedent action representation even in the absence of a real (human, living) actor.

Interestingly, the model and related interventionist accounts make one very clear prediction, which sets them apart from perceptual accounts: Causal learning should be more efficient when human actions and their consequences are observed compared with correlated changes in perceived not-action-related movements. By manipulating the action feature of the perceived antecedent movement (moving objects versus objects moved by a hand), a recent seminal study provided evidence that young children were more likely to draw causal inferences when correlated events were the outcome of human intervention than when they were not [60]. This result supports a role of human body action in causal learning from perception.

## 6. From causal events to causation

Humans and animals are equipped with a mechanism that allows them to map actions on their consequences. The present paper proposes that exactly this mechanism, realized primarily as fronto-posterior cortical connections, provides the basis of their knowledge about causality. However, there is still a gap between knowing that my operating the light switch with the hand makes the bulb light up and the formation of an abstract concept capturing variable causal relationships and generalizing to new instances. A mechanism is needed that binds together the variable instances of causality into a coherent concept.

Evidently, actions can vary substantially and bring about quite different typical effects. The actions of operating the light switch, kicking a ball and blowing a horn involve variable movements of different body parts (finger, foot, mouth) and their effects are manifest in different sensory modalities (visual, somatosensory, acoustic). Given the variability of goal-directed actions, the zero assumption may be that causal actions are entirely different with regard to their cognitive and neurobiological mechanisms. This would mean distinct and non-overlapping causal-action circuits for different actions and their goals. If such *non-overlapping distinctness* is realistic, general knowledge about causation can be represented by a mechanism that computes an EITHER–OR function across all, or at least several, previously encountered and stored instances of causation. Where in the brain might such a binding mechanism be located? Neuroanatomical connectivity is characterized by strong links between adjacent cortical areas and by substantial neuronal divergence and convergence [61,62]. Therefore, next-neighbours of the areas occupied by causal-action circuits appear likely candidates for housing neurons that receive specific input from several such circuits. These neurons would thus respond similarly to a selection of causal events, so that they become *causal key neurons*—not due to correlated neural activity, but because of the partly genetically pre-preprogrammed and partly random neuroanatomical connectivity structure between areas. In this perspective, areas adjacent to those hosting the causal-action circuits would be likely to carry causal key neurons, the putative bricks for building the abstract concept of causation (*adjacent convergence model*). Problems of this approach include its reliance on stochastic connectivity or preprogrammed information, which may appear as too strong a presupposition, and its lack of explanation for systematic generalization of the concept of causation to new instances.

However, there is an alternative mechanism to adjacent convergence, if one questions the non-overlapping distinctness of the broad family of causal event types—and strong arguments can be marshalled in favour of this position. Although causal actions and their effects are very variable, it appears that at least subsets of them share features, which can be motivated neurobiologically. If this is so, the perceptual and action-related features shared by different instances of causation may become key to conceptual binding.

The brisk hand movement towards the mobile, the hitting of a small object and the throwing away of such an object represent quite different basic actions, but ones that share motor features, for example the contraction of the triceps brachii muscle and the sudden application of strong force for a short period of time. It is conceivable that the central nervous system possesses neurons characterized by such motor features (see, for example, [63]), which would thus be activated whenever one of the mentioned causal actions is processed. Likewise, the expected outcomes or goals of mobile-bashing, object-hitting and throwing share perceptual features, namely the perception of an object suddenly moving away from the body; and there are perceptual neurons responding specifically to away movement, irrespective of the nature of the moving object (see, for example, [64]). Thus, the processing of a significant subset of causal body actions will probably be accompanied by activity of overlapping sets of such shared-feature neurons, even though the actions and their goals might differ significantly between each other. There are other subsets of causal actions also sharing sensorimotor features—take, for example, the cases of hitting a billiard ball with a cue, operating a light switch and firing a gun, which all are typically accompanied by a brief sound and followed by a change in the visual field. Most instances of causation share that strong force is briefly applied and abrupt perceptual changes occur, although other cases of causation lack these commonalities (e.g. moving down the blinds by slowly turning a handle). As is the case for other abstract terms, the instances to which the word ‘cause' applies—what it is usually used to speak about—show a pattern of family resemblance (see earlier discussion above and figure 2). In this view, different causal events partly share perceptual, motor and goal-related features so that their neurobiological mechanisms may overlap with regard to part of their underpinning neuronal circuits. Neurons shared by a range of different causal-action circuits—carrying information about abrupt visual–perceptual change, brisk movement onset or the touch of a target object—may thus be key to building the causation concept (*shared grounding model*, [28]). These key neurons shared between causal circuits occupy the same areas across which the latter are distributed—including modality-preferential and modality-general areas such as the motor, premotor and posterior prefrontal cortex, somatosensory cortex and parietal areas posterior to it, and posterior and anterior temporal areas in the ventral ‘what' streams of visual and auditory processing.

**Figure 4.**
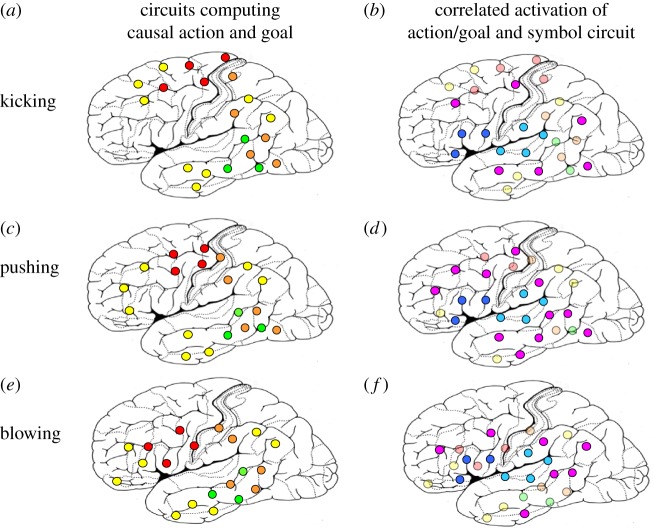
(*a*,*c*,*e*) Differences in the cortical distribution of neuronal assemblies proposed to be involved in processing different causal actions (in red and orange) and their typical effects (in green). Neurons in multimodal convergence zones are also activated as a consequence of causal-action processing (yellow neural elements). Note that some of the neural elements of each distributed circuit are shared with other such circuits (identical location is used to indicate identity). (*b*,*d*,*f*) When it comes to learning (grounding) the word ‘cause' in the context of different causal actions, these shared neural elements (now highlighted in magenta/purple) show the highest correlation with the word form circuit activation and thus become key to the formation of the conceptual circuit.

The model in figure 4 takes into account both possible mechanisms, thus allowing causal key neurons indexing partially shared features of causal actions and their goals (in and close to modality-preferential areas) along with key neurons that are relevant because of random convergence (in multimodal areas only).

## 7. A role for symbols in building the abstract concept

Independent from the cortical localization of the mechanisms of abstraction, the conceptual binding question still needs an answer. How can the different neurocognitive elements—which bind together either random selections of instances of an abstract concept (convergence model) or partially-shared surface sensorimotor and goal-related features of such instances (grounding model)—be joined together to build the abstract concept? I propose that language plays a crucial role here. By using words, or other symbols, in the context of different instances of the abstract concept, the word representation will bind to the causal key neurons, which are activated in variable contexts where the term applies. Note that correlation learning commands that neural elements processing idiosyncratic or atypical features of causal acts are not bound into the circuit. Instead, the neurons indexing shared features of several instances of causation, or convergence neurons that just happen to be linked up with a number of instance representations, show reasonable correlation with the related word form circuit—if the word is used in the context of causal actions. Likewise, if words such as ‘cause' or ‘make (something move in a particular way)' are used in the context of previously grounded action words, indirect grounding by way of shared sensorimotor semantic key neurons may result. Therefore, these key neurons are good candidates for building a circuit integrating the knowledge about the word with that about the variable typical causal-action features, or causal-action subsets. In essence, correlated neuronal activity of partially shared key neurons in relevant modality-related and adjacent convergence areas (purple neuronal units in figure 4, diagrams on the right) together with activity of the word form circuit in the perisylvian cortex (blue units) is the driving force of concept formation. This correlation leads to a cell assembly for the concept of causation, which is distributed across the perisylvian cortex and adjacent multimodal areas but also reaches into sensorimotor fields. The binding of a small number of different sets of semantic key neurons to the same word form may be driven either by direct (word–world feature correlation) or by indirect (word–word correlation) learning.

The result is a distributed neuronal circuit interlinking the word form ‘cause' (or ‘make') with semantic key neurons. Assuming adjacent convergence, these are those neuronal units in close-by areas that just happen to be connected with a relevant subset of causal-action representations (figure 5a). In contrast, the shared grounding model includes semantic key neurons indexing shared and thus typical features of basic actions and their action goals. Note that a main difference lies in the grounding model's extension of the semantic network into sensorimotor fields, which contrasts with the limitation of semantics to multimodal fields of the ‘disembodied' variant.

**Figure 5.**
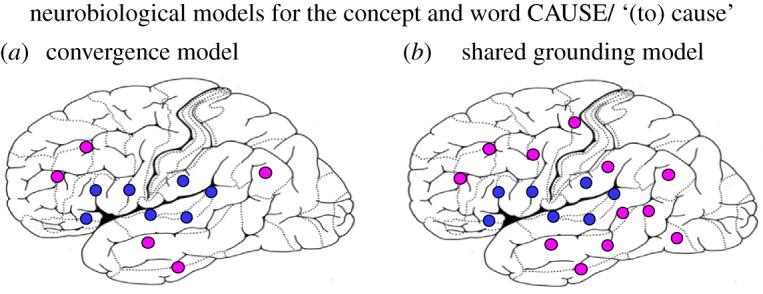
Neurobiological models for the abstract concept of causation. The blue neural elements indicate the neurobiological word form representation and the magenta/purple ones the concept representation. According to the convergence model (*a*), abstract conceptual-semantic neural units are restricted to multimodal areas, whereas the shared grounding model (*b*) places them in both multimodal and modality-preferential areas. For explanation, see text.

## 8. Evidence and outlook

**Figure 6.**
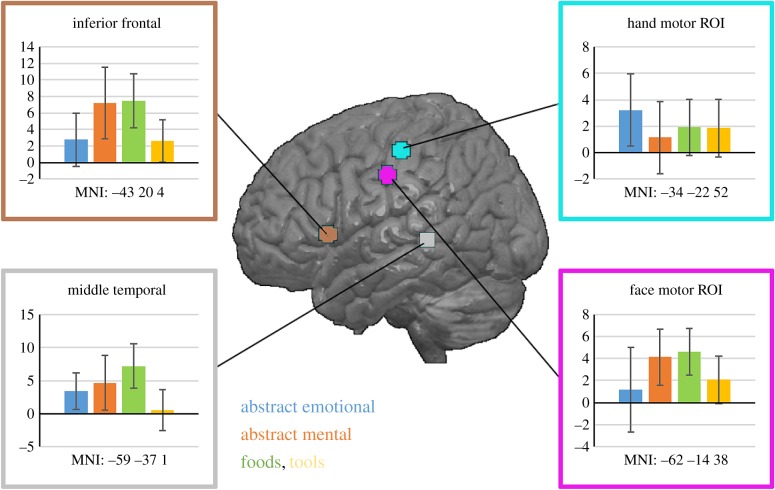
Neurometabolic activity elicited during passive reading of subtypes of abstract and concrete words (abstract emotional and abstract mental nouns in blue and orange; concrete food and tools nouns in green and yellow). Boxes display group average blood-oxygenation-level dependent (BOLD) responses as bars and 95% confidence intervals as whiskers. Significantly different activity patterns for abstract and concrete word subtypes were elicited in sensorimotor areas in the fronto-central cortex (regions of interest (ROIs) indicated in cyan and magenta), but not in the perisylvian language cortex (indicated in grey and brown). Coordinates indicate centre coordinates of ROIs in MNI (Montreal Neurological Institute) space. (Reprinted with permission from [20].)

Current data do not unequivocally decide between the random convergence and shared grounding models. Some results show an involvement of the modality-preferential sensorimotor cortex in abstract word processing (figure 6), thus being consistent with grounding in partially overlapping features of different instances of causal actions (e.g. [16,19,20,65]). Other results speak in favour of a role for modality unspecific areas far from perceptual or motor regions, which seems consistent with a role of neural convergence between areas [66–68]. Likewise, neural activity indexing semantic similarity between words and concepts has been reported in both modality-general and modality-preferential sensorimotor systems [69–72]. As the grounding model, but not the adjacent convergence proposal, captures both types of results, it tends to provide the better fit.

The current perspective is that humans and animals are equipped with a mechanism that allows them to map actions on their consequences. Exactly this mechanism, realized primarily as fronto-posterior cortical connections, provide the basis of their knowledge of causality. This proposal suggests that the knowledge about causality arises from the ‘feeling' (perception, automatic instant interpretation) that an observed event can be brought about or influenced by one's own actions, consistent with the interventionist perspective [73]. This ‘feeling' would not arise for correlated perceptions that are not readily linked with own actions, therefore offering an explanation why pure perceptual correlation is not enough for inferring causal links. Admittedly, this view may not seem intuitive for some cases of non-human causation—for example, a lightning bolt ‘lighting up' a tree—because of the strong implication that even in such cases own body action lays the ground for causal understanding. Therefore, more research is needed to address such claims. One pathway is offered by deprived populations, for example autistic people who typically suffer from an early deficit in motor abilities and, surprisingly, concordant semantic conceptual processing abnormalities (see earlier discussion and [18]).

The model may appear too restricted, as it seemingly applies to only one concept and word—namely CAUSE and ‘cause'—and even one that is usually acquired late in language acquisition. However, there is a family of related words (‘make', ‘let', ‘help', ‘let', ‘hinder', ‘prevent' etc.), which can replace ‘cause' in various contexts (see [34]). And there is an even greater variety of causal action verbs that compositional-semantic theories analyse as including the CAUSE concept (the meaning of ‘kill' analysed as ‘CAUSE to die', [32]). The concept of causation can also be linked to grammatical constructions irrespective of the word forms they contain, for example, to the caused-motion construction [74]. The proposed mechanisms may, therefore, be relevant for a broader variety of linguistic entities. Clearly, the grounding in real-life events of only a few of these concepts is sufficient for allowing indirect, contextual learning of a wider range. But without grounding even one of them directly ‘in the world', it is difficult to explain how an understanding of causation may at all arise.

From a theoretical perspective, shared grounding offers an explanation of generalization, implying that common surface features of typical causal actions and action goals are most likely to make an observed action or perception a candidate for causal interplay. This postulate is, in principle, open to experimental testing. Actions with typical features of causality should be most probably interpreted as causal. If the suggestions offered above are correct, an abruptly starting forceful action should thus be more likely to be perceived as causal than a smooth long-lasting movement. Likewise, intransitive action verbs used to speak about such abrupt forceful actions (for example ‘sneeze', ‘run', ‘jump', ‘startle') would be predicted to be more readily introduced into caused motion constructions (‘She sneezed the foam off the cappuccino'; see [75]) than relatively ‘lame' action verbs (yawn', ‘walk', ‘sleep', ‘relax’, ‘rest’; for example, ‘?He yawned his date out of the restaurant').

The current perspective proposes concrete cognitive and brain mechanisms for at least some typical cases of causation. Not all variants of causation are covered; for example, the intentional variant of making people do something [33] is not covered, because a second action representation would be required in this case. This proposal offers an explanation how one variant of the concept of causation can develop in the mind and brain based on human action and interaction. Cognitivist accounts, according to which abstract concepts are given to us and, by way of unspecified mechanisms, linked up with symbols, ignore and skip this important issue, or consider it as not so important, which seems unsatisfactory. They also fail to account for the well-known fact that abstract concepts vary widely between cultures and language communities. In contrast, differences in the language- and culture-specific social environment and their impact on concepts are taken into account explicitly in the grounding perspective.

By showing for one highly abstract key term that its meaning can be learned and derived from experience, this article does in no way rule out the possibility of a largely genetically preprogrammed basis for other concepts. For example, the logical particles AND, OR and NOT have an obvious analogue in neurons or neuron populations with high or low activation threshold and in inhibitory connections [30,76], thus being rooted in neurophysiology. However, given that a good range of abstract meanings can be explained by a neurobiologically founded grounding model, and given that even highly abstract symbols, such as ‘cause', whose meanings seem far removed from experience, receive a plausible explanation from feature-based grounding mechanisms, there seems to be good reason to consider grounding—both direct and indirect—as an important step towards abstract concept formation.

## Data accessibility

This article has no additional data.

## References

[1] Collins AM, Loftus EF 1975 A spreading activation theory of semantic processing. Psychol. Rev. 82, 407–428. (10.1037/0033-295X.82.6.407)

[2] Landauer TK, McNamara DS, Dennis S, Kintsch W 2013 Handbook of latent semantic analysis. Hove, UK: Psychology Press.

[3] Harnad S 1990 The symbol grounding problem. Physica D 42, 335–346. (10.1016/0167-2789(90)90087-6)

[4] Leshinskaya A, Caramazza A 2016 For a cognitive neuroscience of concepts: moving beyond the grounding issue. Psychon. Bull. Rev. 23, 991–1001. (10.3758/s13423-015-0870-z) 27294420

[5] Mahon BZ 2015 What is embodied about cognition? Lang. Cogn. Neurosci. 30, 420–429. (10.1080/23273798.2014.987791) 25914889 PMC4405253

[6] Wittgenstein L 1953 Philosophical investigations. Oxford, UK: Blackwell Publishers.

[7] Quine WVO 1960 Word and object. Cambridge, MA: MIT Press.

[8] Pulvermüller F 2018 Neural reuse of action perception circuits for language, concepts and communication. Prog. Neurobiol. 160, 1–44. (10.1016/j.pneurobio.2017.07.001) 28734837

[9] Pulvermüller F In press Neurobiological mechanisms for semantic feature extraction and conceptual flexibility. Topics in Cognitive Science. 10.1111/tops.12367 30129710

[10] Cangelosi A, Harnad S 2001 The adaptive advantage of symbolic theft over sensorimotor toil: grounding language in perceptual categories. Evol. Commun. 4, 117–142.

[11] Dove G 2009 Beyond perceptual symbols: a call for representational pluralism. Cognition 110, 412–431. (10.1016/j.cognition.2008.11.016) 19135654

[12] Dove G 2016 Three symbol ungrounding problems: abstract concepts and the future of embodied cognition. Psychon. Bull. Rev. 23, 1109–1121. (10.3758/s13423-015-0825-4) 25832355

[13] Kousta ST, Vigliocco G, Vinson DP, Andrews M, Del Campo E 2011 The representation of abstract words: why emotion matters. J. Exp. Psychol. Gen. 140, 14–34. (10.1037/a0021446) 21171803

[14] Alston WP 1964 Philosophy of language. Englewood Cliffs, NJ: Prentice-Hall.

[15] Baker GP, Hacker PMS 2009 Wittgenstein: understanding and meaning, part 1. Oxford, UK: Wiley-Blackwell.

[16] Moseley R, Carota F, Hauk O, Mohr B, Pulvermüller F 2012 A role for the motor system in binding abstract emotional meaning. Cereb. Cortex 22, 1634–1647. (10.1093/cercor/bhr238) 21914634 PMC3377965

[17] Havas DA, Glenberg AM, Gutowski KA, Lucarelli MJ, Davidson RJ 2010 Cosmetic use of botulinum toxin-A affects processing of emotional language. Psychol. Sci. 21, 895–900. (10.1177/0956797610374742) 20548056 PMC3070188

[18] Moseley RL, Pulvermüller F 2018 What can autism teach us about the role of sensorimotor systems in higher cognition? New clues from studies on language, action semantics, and abstract emotional concept processing. Cortex 100, 149–190. (10.1016/j.cortex.2017.11.019) 29306521

[19] Borghi AM, Zarcone E 2016 Grounding abstractness: abstract concepts and the activation of the mouth. Front. Psychol. 7, 1498. 27777563 10.3389/fpsyg.2016.01498 PMC5056183

[20] Dreyer FR, Pulvermüller F 2018 Abstract semantics in the motor system?–An event-related fMRI study on passive reading of semantic word categories carrying abstract emotional and mental meaning. Cortex 100, 52–70. (10.1016/j.cortex.2017.10.021) 29455946

[21] Barsalou LW 1999 Perceptual symbol systems. Behav. Brain Sci. 22, 577–660. 11301525 10.1017/s0140525x99002149

[22] Barsalou LW, Wiemer-Hastings K 2005 Situating abstract concepts. In Grounding cognition: the role of perception and action in memory, language, and thought (eds Pecher D, Zwaan R), pp. 129–163. New York, NY: Cambridge University Press.

[23] Stramandinoli F, Marocco D, Cangelosi A 2017 Making sense of words: a robotic model for language abstraction. Auton. Robots 41, 367–383. (10.1007/s10514-016-9587-8)

[24] Della Rosa PA, Catricalà E, Vigliocco G, Cappa SF 2010 Beyond the abstract–concrete dichotomy: mode of acquisition, concreteness, imageability, familiarity, age of acquisition, context availability, and abstractness norms for a set of 417 Italian words. Behav. Res. Methods 42, 1042–1048. (10.3758/BRM.42.4.1042) 21139171

[25] Altarriba J, Bauer LM, Benvenuto C 1999 Concreteness, context availability, and imageability ratings and word associations for abstract, concrete, and emotion words. Behav. Res. Methods Instrum. Comput. 31, 578–602. (10.3758/BF03200738) 10633977

[26] Nelson DL, Schreiber TA 1992 Word concreteness and word structure as independent determinants of recall. J. Mem. Lang. 31, 237–260. (10.1016/0749-596X(92)90013-N)

[27] Rosch E, Mervis CB 1975 Family resemblances: studies in the internal structure of categories. Cognit. Psychol. 7, 573–605. (10.1016/0010-0285(75)90024-9)

[28] Pulvermüller F 2013 How neurons make meaning: brain mechanisms for embodied and abstract-symbolic semantics. Trends Cogn. Sci. 17, 458–470. (10.1016/j.tics.2013.06.004) 23932069

[29] Schwanenflugel P 1991 Why are abstract concepts hard to understand? In The psychology of word meaning (ed. Schwanenflugel PJ), pp. 223–259. Hillsdale, NJ: Lawrence Erlbaum Associates.

[30] McCulloch WS, Pitts WH 1943 A logical calculus of ideas immanent in nervous activity. Bull. Math. Biophys. 5, 115–133. (10.1007/BF02478259) 2185863

[31] Gopnik A, Glymour C, Sobel DM, Schulz LE, Kushnir T, Danks D 2004 A theory of causal learning in children: causal maps and Bayes nets. Psychol. Rev. 111, 3–32. (10.1037/0033-295X.111.1.3) 14756583

[32] Jackendoff R 2002 Foundations of language: brain, meaning, grammar, evolution. Oxford, UK: Oxford University Press. 10.1017/s0140525x03000153 15377127

[33] von Wright GH 1971 Explanation and understanding. Ithaca, NY: Cornell University Press.

[34] Talmy L 1988 Force dynamics in language and cognition. Cogn. Sci. 12, 49–100. (10.1207/s15516709cog1201_2)

[35] Pulvermüller F 2008 Grounding language in the brain. In Symbols, embodiment, and meaning (eds de Vega M, Graesser A, Glenberg AM), pp. 85–116. Oxford, UK: Oxford University Press.

[36] Hume D 2007 An enquiry concerning human understanding. Oxford, UK: Oxford University Press.

[37] Baldwin D, Andersson A, Saffran J, Meyer M 2008 Segmenting dynamic human action via statistical structure. Cognition 106, 1382–1407. (10.1016/j.cognition.2007.07.005) 18035346

[38] Roseberry S, Richie R, Hirsh-Pasek K, Golinkoff RM, Shipley TF 2011 Babies catch a break: 7- to 9-month-olds track statistical probabilities in continuous dynamic events. Psychol. Sci. 22, 1422–1424. (10.1177/0956797611422074) 22020978

[39] Stahl AE, Romberg AR, Roseberry S, Golinkoff RM, Hirsh-Pasek K 2014 Infants segment continuous events using transitional probabilities. Child Dev. 85, 1821–1826. (10.1111/cdev.12217) 24749627 PMC4165826

[40] Buchsbaum D, Griffiths TL, Plunkett D, Gopnik A, Baldwin D 2015 Inferring action structure and causal relationships in continuous sequences of human action. Cogn. Psychol. 76, 30–77. (10.1016/j.cogpsych.2014.10.001) 25527974

[41] Saffran JR, Aslin RN, Newport EL 1996 Statistical learning by 8-month-old infants. Science 274, 1926–1928. (10.1126/science.274.5294.1926) 8943209

[42] Piaget J 1930 The child's conception of physical causality. New York, NY: Harcourt Brace.

[43] Piaget J 1954 The construction of reality in the child. New York, NY: Basic Books.

[44] Watson JS, Ramey CT 1972 Reactions to response-contingent stimulation in early infancy. Merrill-Palmer Quart. 18, 219–227.

[45] Sommerville JA, Woodward AL, Needham A 2005 Action experience alters 3-month-old infants' perception of others’ actions. Cognition 96, B1–B11. (10.1016/j.cognition.2004.07.004) 15833301 PMC3908452

[46] Libertus K, Needham A 2010 Teach to reach: the effects of active vs. passive reaching experiences on action and perception. Vis. Res. 50, 2750–2757. 20828580 10.1016/j.visres.2010.09.001 PMC2991490

[47] Rakison DH, Krogh L 2012 Does causal action facilitate causal perception in infants younger than 6 months of age? Dev. Sci. 15, 43–53. (10.1111/j.1467-7687.2011.01096.x) 22251291

[48] Haruno M, Wolpert DM, Kawato M 2003 Hierarchical MOSAIC for movement generation. International Congress Series Amsterdam, The Netherlands: Elsevier.

[49] Fuster JM, Bressler SL 2012 Cognit activation: a mechanism enabling temporal integration in working memory. Trends Cogn. Sci. 16, 207–218. (10.1016/j.tics.2012.03.005) 22440831 PMC3457701

[50] Fuster JM 2001 The prefrontal cortex—an update: time is of the essence. Neuron 30, 319–333. (10.1016/S0896-6273(01)00285-9) 11394996

[51] Waismeyer A, Meltzoff AN, Gopnik A 2015 Causal learning from probabilistic events in 24-month-olds: an action measure. Dev. Sci. 18, 175–182. (10.1111/desc.12208) 25041264

[52] Danto AC 1965 Basic actions. Am. Philos. Q. 2, 141–148.

[53] Rizzolatti G, Craighero L 2004 The mirror-neuron system. Ann. Rev. Neurosci. 27, 169–192. (10.1146/annurev.neuro.27.070203.144230) 15217330

[54] Rizzolatti G, Sinigaglia C 2016 The mirror mechanism: a basic principle of brain function. Nat. Rev. Neurosci. 17, 757–765. (10.1038/nrn.2016.135) 27761004

[55] Pulvermüller F 1999 Words in the brain's language. Behav. Brain Sci. 22, 253–336. (10.1017/S0140525X9900182X) 11301524

[56] Keysers C, Perrett DI 2004 Demystifying social cognition: a Hebbian perspective. Trends Cogn. Sci. 8, 501–507. (10.1016/j.tics.2004.09.005) 15491904

[57] Gopnik A, Schulz L (eds). 2007 Causal learning: psychology, philosophy, and computation. New York, NY: Oxford University Press.

[58] Woodward J 2015 Interventionism and causal exclusion. Philos. Phenomenol. Res. 91, 303–347. (10.1111/phpr.12095)

[59] Woodward J 2007 Interventionist theories of causation in psychological perspective. In Causal learning: psychology, philosophy, and computation (eds Gopnik A, Schulz L), pp. 19–36. New York, NY: Oxford University Press.

[60] Meltzoff AN, Waismeyer A, Gopnik A 2012 Learning about causes from people: observational causal learning in 24-month-old infants. Dev. Psychol. 48, 1215–1228. (10.1037/a0027440) 22369335 PMC3649070

[61] Damasio AR 1989 The brain binds entities and events by multiregional activation from convergence zones. Neural Comput. 1, 123–132. (10.1162/neco.1989.1.1.123)

[62] Braitenberg V, Schüz A 1998 Cortex: statistics and geometry of neuronal connectivity, 2nd edn Berlin, Germany: Springer.

[63] Crutcher MD, Alexander GE 1990 Movement-related neuronal activity selectively coding either direction or muscle pattern in three motor areas of the monkey. J. Neurophysiol. 64, 151–163. (10.1152/jn.1990.64.1.151) 2388062

[64] Bruce C, Desimone R, Gross CG 1981 Visual properties of neurons in a polysensory area in superior temporal sulcus of the macaque. J. Neurophysiol. 46, 369–384. (10.1152/jn.1981.46.2.369) 6267219

[65] Glenberg AM, Sato M, Cattaneo L, Riggio L, Palumbo D, Buccino G 2008 Processing abstract language modulates motor system activity. Q. J. Exp. Psychol. 61, 905–919. (10.1080/17470210701625550) 18470821

[66] Ghio M, Vaghi MMS, Perani D, Tettamanti M 2016 Decoding the neural representation of fine-grained conceptual categories. Neuroimage 132, 93–103. (10.1016/j.neuroimage.2016.02.009) 26883065

[67] Wilson-Mendenhall CD, Simmons WK, Martin A, Barsalou LW 2013 Contextual processing of abstract concepts reveals neural representations of nonlinguistic semantic content. J. Cogn. Neurosci. 25, 920–935. (10.1162/jocn_a_00361) 23363408 PMC3947606

[68] Binder JR, Westbury CF, McKiernan KA, Possing ET, Medler DA 2005 Distinct brain systems for processing concrete and abstract concepts. J. Cogn. Neurosci. 17, 905–917. (10.1162/0898929054021102) 16021798

[69] Carota F, Kriegeskorte N, Nili H, Pulvermuller F 2017 Representational similarity mapping of distributional semantics in left inferior frontal, middle temporal, and motor cortex. Cereb. Cortex 27, 294–309. 28077514 10.1093/cercor/bhw379 PMC6044349

[70] Carlson TA, Simmons RA, Kriegeskorte N, Slevc LR 2014 The emergence of semantic meaning in the ventral temporal pathway. J. Cogn. Neurosci. 26, 120–131. (10.1162/jocn_a_00458) 23915056

[71] Fairhall SL, Caramazza A 2013 Brain regions that represent amodal conceptual knowledge. J. Neurosci. 33, 10 552–10 558. (10.1523/JNEUROSCI.0051-13.2013) PMC6618586 23785167

[72] Quandt LC, Lee YS, Chatterjee A 2017 Neural bases of action abstraction. Biol. Psychol. 129, 314–323. (10.1016/j.biopsycho.2017.09.015) 28964789 PMC5673573

[73] Woodward J 2005 Making things happen: a theory of causal explanation. New York, NY: Oxford University Press.

[74] Goldberg AE 1995 Constructions: a construction grammar approach to argument structure. Chicago, IL: University of Chicago Press.

[75] Goldberg AE 2006 Constructions at work: the nature of generalisation in language. Oxford, UK: Oxford University Press.

[76] Kleene SC 1956 Representation of events in nerve nets and finite automata. In Automata studies (eds Shannon CE, McCarthy J), pp. 3–41. Princeton, NJ: Princeton University Press.

